# Mapping atypical UV photoproducts *in vitro* and across the *S. cerevisiae* genome

**DOI:** 10.1016/j.xpro.2021.101059

**Published:** 2021-12-22

**Authors:** Kaitlynne A. Bohm, Smitha Sivapragasam, John J. Wyrick

**Affiliations:** 1 School of Molecular Biosciences, Washington State University, Pullman, WA 99164, USA; 2 Center for Reproductive Biology, Washington State University, Pullman, WA 99164, USA

**Keywords:** Bioinformatics, Model Organisms, Molecular Biology, Sequence analysis, Sequencing

## Abstract

Exposure to ultraviolet (UV) light induces DNA damage, predominantly cyclobutane pyrimidine dimers (CPD) and 6,4-photoproducts (6,4-PP), as well as rare, atypical photoproducts at thymidine-adenine (TA) sequences. We have recently shown ‘TA’ photoproducts are induced in UV-irradiated oligonucleotides and across the budding yeast genome. Here, we describe a protocol for mapping atypical ‘TA’ photoproducts *in vitro* and *in vivo.* This protocol overcomes the technical challenges involved in accurately mapping such rare photoproducts by using ultraviolet damage endonuclease (UVDE) enzymes.

For complete details on the use and execution of this protocol, please refer to Laughery et al. (2020).

## Highlights

• UVDE enzyme cleaves ‘TA’ photoproducts in UV-irradiated oligos and the yeast genome
• CPD photolyase treatment ensures only 6,4-PPs and atypical lesions are mapped
• Bioinformatic pipelines precisely locate where these ‘TA’ lesions formed *in vivo*

## Graphical Abstract

**Figure:**
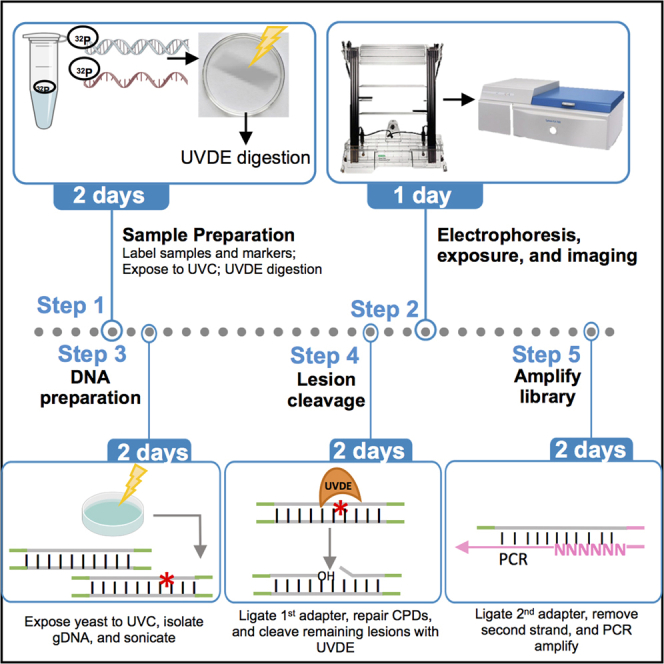

## Before you begin

**Figure 1:**
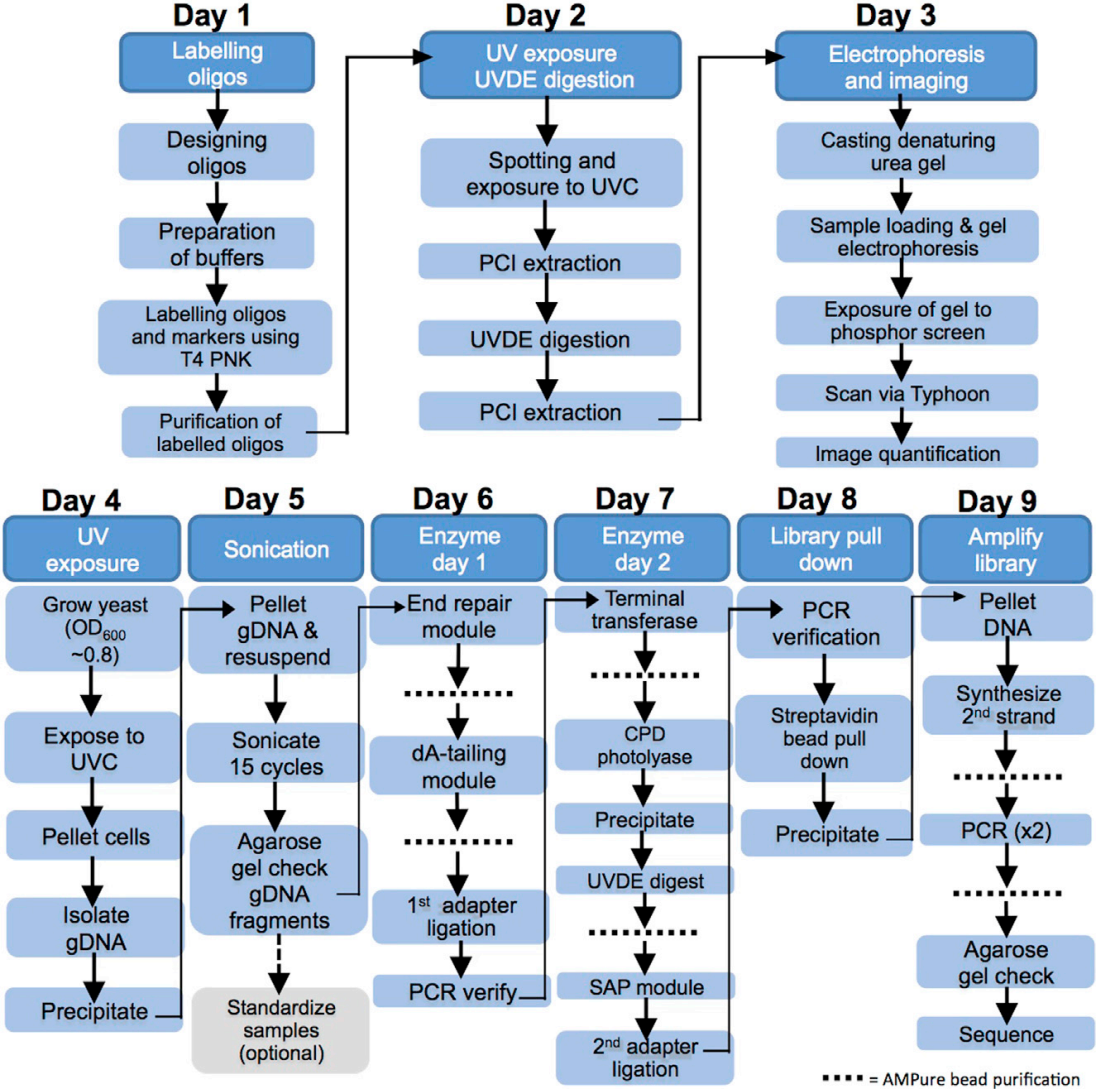
*In vitro* and *in vivo* ‘TA’ photoproduct cleavage protocol overview

Here we describe a protocol that utilizes the recently discovered ability of UVDE to cleave atypical ‘TA’ UV lesions. The *in vitro* section of the protocol validates UVDE cleavage of ‘TA’ photoproducts in oligonucleotides, and the *in vivo* UVDE-seq section of the protocol uses UVDE to map atypical photoproducts throughout the *Saccharomyces cerevisiae* genome (Figure 1). This protocol may be modified for use in other organisms or cell lines.

### Design primers

**Timing: 0.5 days**

**Figure 2:**
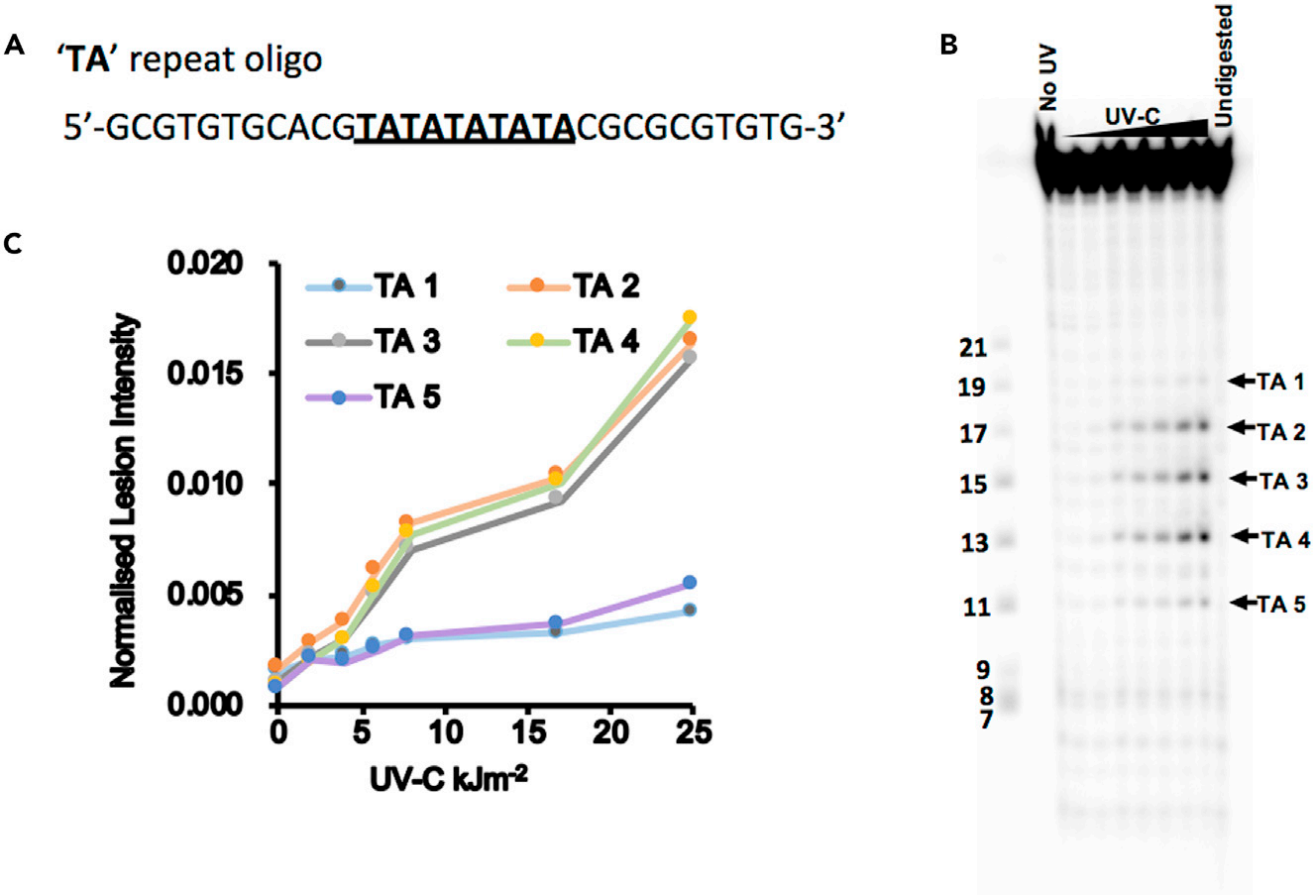
*In vitro* characterization of ‘TA’ photoproduct (adapted from Laughery et al., 2020)

1. Design oligos containing five ‘TA’ repeats (Figure 2) and the marker oligos spanning the expected sizes of ‘TA’ lesions, as well as adapters and primers for *in vivo* mapping.

### Prep equipment and reagents

**Timing: 1 day**

2. Wipe down the beta shield, equipment (gel apparatus), and goggles using ethanol to clear away dirt/dust and to have a better visibility.
3. Prepare buffers and reagents essential for the experiment and plan using ^32^P within the first half-life for best signal.
4. Adapters must be ligated prior to use. To do this, combine 40 μL of 100 mM of each strand (top and bottom, Table 1), 10 μL 10× ligase buffer, and 10 μL dH_2_O. Incubate using the annealing conditions below.

**Table 1:** Oligonucleotides used for UVDE-seq

**Adapters**	
trP1 top	5′-CCTCTCTATGGGCAGTCGGTGAT-phosphorothioate-T-3′
trP1 bottom	5′-phosphate-ATCACCGACTGCCCATAGAGAGGC-dideoxy-3′
A1 top	5′-phosphate-ATCCTCTTCTGAGTCGGAGACACGCAGGGATGAGATGGC-dideoxy-3′
A1 bottom	5′-biotin-CCATCTCATCCCTGCGTGTCTCCGACTCAGAAGAGGATNNNNNN-C3 phosphoramidite-3′
A2 top	5′-phosphate-ATCACGAACTGAGTCGGAGACACGCAGGGATGAGATGGC-dideoxy-3′
A2 bottom	5′-biotin-CCATCTCATCCCTGCGTGTCTCCGACTCAGTTCGTGATNNNNNN-C3 phosphoramidite-3′
A3 top	5′-phosphate-ATCTCAGGCTGAGTCGGAGACACGCAGGGATGAGATGGC-dideoxy-3′
A3 bottom	5′-biotin-CCATCTCATCCCTGCGTGTCTCCGACTCAGCCTGAGATNNNNNN-C3 phosphoramidite-3′
A4 top	5′-phosphateATCGCGATCTGAGTCGGAGACACGCAGGGATGAGATGGC-dideoxy-3′
A4 bottom	5′- biotin-CCATCTCATCCCTGCGTGTCTCCGACTCAGATCGCGATNNNNNN-C3 phosphoramidite-3′

**Primers**	

Primer trP1	5′-CCTCTCTATGGGCAGTCGGTGATT-3′
Primer A	5′-CCATCTCATCCCTGCGTGTCTCCGAC-3′

**Table:** 

Steps	Temperature	Time
Initial denaturation	95°C	5 min
Annealing	95°C	1 min
−1°C per cycle		
Hold	4°C	Forever

5. 1.5M NaOH must be made fresh, either once a month or after every 3 uses, whichever comes first. 0.15M NaOH must be made fresh the day it will be used.

## Key resources table

**Table:** 

REAGENT or RESOURCE	SOURCE	IDENTIFIER
**Chemicals, peptides, and recombinant proteins**		

*E. coli* CPD photolyase	Dr. Steven Roberts (Laughery et al., 2020)	N/A
*T. Thermophilus* UVDE enzyme	Dr. Steven Roberts (Laughery et al., 2020)	N/A

**Critical commercial assays**		

Ampure XP Beads	Beckman Coulter	A63881
ATP(γ-^32^P)	PerkinElmer	BLU002Z250UC SB4
Dynabeads M-280 Streptavadin beads	Thermo Scientific	11205D
Glycogen	Thermo Fisher Scientific	FERR0561
Next dA-Tailing Module	New England Biolabs	E6053L
Next End Repair Module	New England Biolabs	E6050L
Quick Ligase Module	New England Biolabs	E6056L
RNase A	Fisher Scientific	FEREN0531
Shrimp Alkaline Phosphatase	Thermo Fisher Scientific	78390500UN
T4 Polynucleotide Kinase	New England Biolabs	M0201S
Taq polymerase	VWR	2428-9844
Terminal Transferase Module	New England Biolabs	M0315L

**Deposited data**		

Raw UVDE-seq data	This study (Laughery et al., 2020)	GEO: GSE144679

**Experimental models: Organisms/strains**		

*Saccharomyces cerevisiae* (BY4741)	ATCC	4040002

**Oligonucleotides**		

‘TA’ oligos, marker oligos, trP1 and A adapters, and primers (see Tables 1 and Table 2)	Integrated DNA Technologies	N/A

**Software and algorithms**		

Bowtie2	(Langmead and Salzberg, 2012)	http://bowtie-bio.sourceforge.net/bowtie2/index.shtml
Samtools	(Li et al., 2009)	http://samtools.sourceforge.net/

**Other**		

100 bp DNA ladder	New England Biolabs	N3231
100% Ethanol	Decon Labs	V1016
100% Formamide	Fisher Scientific	46-101-00ML
40% Acrylamide: Bisacrylamide	Bio-Rad Laboratories	1610145
5” × 7” plastic developing tray	United States Plastic Corp.	52050
6× purple gel loading dye	New England Biolabs	B7024S
Acid-washed beads	Sigma-Aldrich	G9268-500G
Agarose	Fisher Scientific	BP160-100
Agarose gel apparatus	Thermo Fisher Scientific	09-528-110
Benchtop upright beta shield	FISHER BIOTEC	FB-BT-1-FIS
Beta containers/discards	FISHER BIOTEC	S29138
Beta shields	Nalgene	6700-2418
BioRupter Sonicator	Diagenode, Inc.	UCD-300 TM
Coverslips	Corning	48397-163
ddATP or ddGTP	Roche Diagnostics	03 732 738 001
dNTPs	New England Biolabs	N0447S
Filterpaper sheets	VWR	47749-838
Geiger Muller Counter & probe 44-9	Ludlum	N/A
Gel dryer attached to vacuum	Bio-Rad Laboratories	165-1745/165-1746
Hand-held UVA lamp	Spectroline	ENF-240C
HydroTech Vacuum pump	Bio-Rad Laboratories	Model 583
Illustra G-50 columns	GE Healthcare	27-5330-01
Imagequant TL v8.1 software	Cytiva Life Sciences	29000605
Magnetic phosphor screen	GE healthcare	N/A
Magnetic tube rack	Thermo Fisher Scientific	12321D
Microcentrifuge	Eppendorf	5405000107
NanoDrop Spectrophotometer ND-1000	Thermo Fisher Scientific	SKU: 2353-30-0010
Phenol:Chloroform:Isoamyl alcohol (PCI)	Fisher Scientific	BP1752I-400
Rotator	Express Lab Werks	Tc7
Centrifuge (Sorvall Legend T/RT)	Kendro Laboratory Products	N/A
Sequencing gel apparatus	Bio-Rad Laboratories	165-3860/165-3861/165-3862/165-3863
Stratalinker UV Crosslinker TM1800	StrateGene	400071 (120 V) / 400072 (230 V) / 400672 (100 V)
Thermocycler	Eppendorf	2231000814
TPX tubes	Diagenode, Inc.	C30010010-100 (M-50001)
Typhoon FLA 7000 series	GE Healthcare	N/A
UV light box	N/A	N/A
UV-A Radiometer	Fisher Scientific	06-662-65
UV-C Radiometer	Spectroline	DM-254N

## Materials and equipment

**Table:** 10× TBE buffer

Reagent	Final concentration	Amount
Tris base	0.89M	108 g
Boric Acid	0.89M	55 g
Disodium EDTA	0.02M	9.3 g
dH_2_O up to		1 L

Stir and adjust pH to 8. Store at 23°C for 1+ year.

**Table:** 2× Gel Loading dye

Reagent	Final concentration	Amount
Formamide	95%	9.5 mL
Disodium EDTA	0.5%	50 mg
Xylene cyanol	0.1%	10 mg
Bromophenol blue	0.1%	10 mg
Total		10 mL

Aliquot and store at −20°C for 1+ year.

**Table:** Yeast extract peptone dextrose (YPD) medium

Reagent	Amount
Bacto-peptone	18 g
Yeast extract	9 g
Glucose	18 g
Tryptophan	0.135 g
ddH_2_O	900 mL

Autoclave to sterilize. Store at 23°C for 1+ year.

**Table:** Yeast Lysis buffer

Reagent	Final concentration	Amount
TritonX-100	2% (v/v)	1 mL
SDS 10%	1% (w/v)	5 mL
Tris-Cl 1M pH 8	10mM	0.5 mL
NaCl 5M	100mM	1 mL
EDTA 0.5M pH 8	1mM	0.1 mL
dH_2_O up to		50 mL

Store at 23°C for 1+ year.

**Table:** TE buffer pH 8

Reagent	Final concentration	Amount
Tris-HCl	10 mM	1.57 g
EDTA	1 mM	0.292 g
dH_2_O up to		1 L

Stir and adjust pH to 8. Store at 23°C for 1+ year.

**Table:** 1× Saline sodium citrate (SSC)

Reagent	Final concentration	Amount
3M NaCl	150 mM	2.5 mL
300mM Sodium citrate	15 mM	2.5 mL
dH_2_O up to		50 mL

Store at 23°C for 1+ year.

**Table:** 40% PEG8000

Reagent	Final concentration	Amount
PEG8000	0.4×	4 g
5M NaCl	1.25M	2.5 mL
dH_2_O up to		10 mL

Sterilize. Store at 23°C for up to 6 months.

**Table:** 10× UVDE reaction buffer

Reagent	Final concentration	Amount
200 mM HEPES (pH 6.5)	20 mM	10 mL
1M NaCl	100 mM	10 mL
10mM MnCl_2_	1 mM	10 mL
dH_2_O up to		100 mL

Stir and heat to mix. Filter sterilize. Store at 4°C for one year.

**Table:** CPD Photolyase reaction buffer (Liu et al., 2011)

Reagent	Final concentration	Amount
1M NaCl	100 mM	10 mL
0.5M Tris-HCl (pH 7.5)	50 mM	10 mL
200mM DTT	20 mM	10 mL
10mM EDTA	1 mM	10 mL
100% glycerol	50% (v/v)	50 mL
dH_2_O up to		100 mL

Stir and heat to mix. Filter sterilize. Store at 4°C for one year.

**Table:** 2× Bind and Wash buffer

Reagent	Final concentration	Amount
1M Tris 7.5	20 mM	1 mL
5M NaCl	4M	40 mL
0.5M EDTA	2 mM	200 μL
dH_2_O up to		50 mL

Filter sterilize. Store at 23°C for 1+ year.

**Table:** 3M NaOAc

Reagent	Final concentration	Amount
NaOAc	3M	24.61 g
dH_2_O up to		50 mL

Adjust pH to 5.2 using glacial acetic acid. Store at 23°C for 1+ year.

**Table:** 1.5M NaOH

Reagent	Final concentration	Amount
NaOH	1.5M	0.6 g
dH_2_O up to		10 mL

Store at 23°C for up to one month.

**Table:** 5M NaCl

Reagent	Final concentration	Amount
NaCl	5M	292 g
dH_2_O up to		1 L

Store at 23°C for 1+ year.

**Table:** 0.5M EDTA

Reagent	Final concentration	Amount
Disodium EDTA.2H_2_O	0.5M	186.1 g
dH_2_O up to		1 L

Stir and adjust pH to 8 using NaOH. Store at 23°C for 1+ year.

## Step-by-step method details

**CRITICAL:** Appropriate safety protocol has to be followed while handling radioactive material. Wear Lab coat, goggles and gloves. Each step is performed behind the shield without accidently getting exposed to the beta rays of ^32^P ideally in a radioactive certified hood. Discard the pipette tips carefully into a radioactive waste container.

### Labelling DNA using ^32^P and purification of labelled DNA

**Timing: 1.5–2 h depending upon number of samples to be labelled**

In this step the ‘TA’ oligos and markers (Table 2) are labelled using ATP(γ-^32^P) and purified using G-50 columns to get rid of the unincorporated radioactive nucleotides.

1. Add the ingredients listed below and incubate the reaction mixture at 37°C for 45 min and 65°C for 20 min.

**Table:** 

Reagent	Final concentration	Amount
Annealed Primers (100 μM)	2 μM	1 μL
ATP(γ-^32^P)	0.2 microcurie	1 μL
10× PNK buffer	1×	5 μL
T4 polynucleotide kinase	0.2 units	1 μL
ddH_2_O	N/A	42 μL
Total		50 μL

**Table 2:** Oligonucleotides for *in vitro* UVDE digest

**Annealed primers**	
OWY395	5′-GCGTGTGCACGTATATATATACGCGCGTGTG-3′
OWY396	5′Biotin-CACACGCGCGTATATATATACGTGCACACGC-3′

**‘TA’ oligonucleotide markers**	

OSS27: TA-PP marker oligo, 11 bases	GCGTGTGCACG
OSS28: TA-PP marker oligo, 13 bases	GCGTGTGCACGTA
OSS29: TA-PP marker oligo, 15 bases	GCGTGTGCACGTATA
OSS30: TA-PP marker oligo, 17 bases	GCGTGTGCACGTATATA
OSS31: TA-PP marker oligo, 19 bases	GCGTGTGCACGTATATATA
OSS32: TA-PP marker oligo, 21 bases	GCGTGTGCACGTATATATATA
OSS33: TA-PP marker oligo, 9 bases	GCGTGTGCA
OSS34: TA-PP marker oligo, 8 bases	GCGTGTGC
OSS35: TA-PP marker oligo, 7 bases	GCGTGTG

2. Purify the labelled primers using G-50 columns (GE health care) according to manufacturer’s instruction (illustra MicroSpin G-50 Columns).
  a. Vortex the sephadex G-50 column at low speed to resuspend the column. The tip below should be snipped off and the cap has to be opened to 1/4^th^ turn. Do not open too much to avoid the lid coming off during centrifugation.
  b. Place the column into a collection tube and centrifuge at 735×*g* for one minute.
    ***Note:*** Centrifugation speed and time must be followed strictly according to manufacturer’s recommendation to elute the right amount of DNA. Especially because low speed will lead to inefficient removal of buffer in the first step leading to dilution of purified DNA sample in the elution step. Sometimes, the column might retain the buffer after a single centrifugation. Here it may be necessary to modify the manufacturer’s protocol by an additional centrifugation step for a minute to efficiently remove buffer suspended in the column. Phenol:Chloroform extraction of DNA could be used to purify labelled DNA as an alternative to G-50 column purification.
  c. After packing resin column, add labelled DNA to the center without poking into the resin. Centrifuge at 735×*g* for 2 min to elute labelled DNA.
    ***Note:*** Check the radioactivity of the eluted DNA using a Geiger Muller counter at this step with maximal setting (100×). A stronger signal should be detected usually in the range of 100× or 10×. Failure of labelling or purification steps might lead to weaker signal of labelled samples.

3. Set up a labelling reaction using the marker oligos as listed below.

**Table:** 

Reagent	Amount
Marker oligos OSS35, OSS33, OSS27	17 μL each
Marker oligo OSS34	20 μL
Marker oligos OSS28	7 μL
Marker oligos OSS29, OSS30, OSS31, OSS32	2 μL each
10× PNK buffer	10 μL
ATP(γ^32^-P)	2 μL
Polynucleotide Kinase	2 μL
Total	100 μL

4. Incubate the reaction at 37°C for 45 min and then inactivate the enzyme by incubating at 65°C for 20 min.
5. Purify the labelled oligos using G-50 column as mentioned above.

***Note:*** Some of the base markers with a very small size difference might run closely together on the gel, hindering the exact determination of size. To circumvent this, it is recommended to label those markers separately and load them in a separate well next to the samples. Since G-50 columns cannot efficiently elute oligos less than 20 nucleotides, it is wise to start with comparatively higher amounts of lower size oligonucleotides (at least double the amount of higher size oligonucleotides).

### Exposing labelled oligos to varying doses of UV-C

**Timing: 3–4 h depending upon number of samples**

In this step, the labelled and purified ‘TA’ oligo is exposed to increasing doses of UV-C.

6. Dilute the labelled product from step 5 with ultrapure water to 400 μL. Clean a coverslip with Kimwipe and using a marker pen, draw lines at the back of the cover slip to demarcate samples while spotting.
  a. For each UV exposure, as well as the ‘no exposure’ sample, prepare a petri plate with a cut piece of wet paper towel (cold water). On the back of the clean coverslip draw vertical lines to demarcate 3–4 compartments for samples.
  b. DNA is spotted (as 4 spots of 10 μL) into the allotted spaces and the coverslip is placed into the petri plate.
7. Set the Stratagene UV-C crosslinker in the timer mode. Place the petri plate with the samples inside the UV-C crosslinker. Samples are exposed to UV for different time points namely 2.5 min, 5 min, 7.5 min, 10 min, 12.5 min, 15 min and 30 min.

***Note:*** The wet paper towel minimizes water evaporation from the sample during exposure and helps to avoid sliding of coverslip to the bottom of petri plate.

8. Pool the 10 μL spots after UV exposure at each time point using a pipette and transfer into a 1.5 mL tube. Place tubes on ice until all time point samples have been collected.
9. Add 60 μL of Phenol:Chloroform:Isoamyl alcohol (neutral pH) to the samples and vortex for 30 s. Add 220 μL of water and briefly vortex. Centrifuge the samples at 16,873×*g* for 6 min.
10. Carefully pipette 200 μL of the upper aqueous phase and transfer to a new 1.5 mL Eppendorf tube. Add 3 μL of glycogen, 20 μL of 3M NaOAc pH 5.2 and 800 μL of 100% ice cold ethanol. Mix well and incubate at −20°C for at least 1 h.

**Pause point:** Incubation step could be extended to 16 h at −20°C.

11. Pellet the DNA by centrifuging at highest speed 16,873×*g* for 1 h. The pellet will be visible at the bottom of the tube as a small translucent spot.
12. Pipette out ethanol and add 70% ethanol into each tube. Leave the pellet untouched and centrifuge again for 20 min.
13. Carefully pipette out ethanol without touching the pellet. Do a short spin to get all residual ethanol to the bottom. Carefully pipette out the ethanol left out around the pellet without disturbing the pellet. Let the pellet dry for 5–10 min in the hood.

***Note:*** It is ideal to cast the gel during the day of UVDE digestion.

### UVDE digestion and sample preparation

**Timing: 3–4 h depending upon number of samples**

In this step, the DNA samples exposed to UV are digested with UVDE enzyme and prepared for the next electrophoresis step.

14. Add 1 μL of 10× UVDE buffer (pH 6.5), 8 μL of water and resuspend the pellet. Vortex to mix and centrifuge shortly to bring the reaction mixture to the bottom of the tube.
15. Add 1 μL of UVDE enzyme and incubate at 55°C for 1 h.
  a. Using a pipette mix the enzyme with the reaction mixture before incubating.
16. After incubation add 50 μL of TE buffer (pH 8) and add 60 μL of PCI. Add 250 μL of water and vortex well for 30 s.
17. Centrifuge for 6 min at 16,873×*g*. Carefully pipette out 200 μL of the upper phase and transfer to a new 1.5 mL Eppendorf tube. Add 3 μL of glycogen, 20 μL of 3M sodium acetate pH 5.2 and 800 μL of 100% ethanol and invert the tube few times to mix, then store at −20°C for at least 1 h.

***Note:*** pH neutralization of the sample after UVDE digestion is essential at this step. If not done, this might lead to uneven migration of sample during electrophoresis that is usually carried out under alkaline conditions.

**Pause point:** The incubation here can be extended to 16 h at −20°C.

18. Centrifuge samples at 16,873×*g* for 1 h to pellet DNA. DNA will appear as a small translucent spot at the bottom of the tube.

***Note:*** Set up the gel apparatus and start pre-running the gel during pelleting and washing steps. Check section ‘pre-running, sample loading and gel electrophoresis’ for complete details.

19. Without touching the pellet, carefully pipette out ethanol, add 1 mL of 70% ethanol to the tube and centrifuge at 16,873×*g* for 20 min. Carefully pipette out ethanol. Do a short spin to collect residual ethanol sticking on to the sides of the tube. Using a pipette carefully pipette out residual ethanol without touching the pellet.

***Note:*** Once DNA is pelleted using 100% ethanol, leave the pellet undisturbed in the subsequent steps during 70% ethanol wash or do not resuspend the pellet during ethanol wash step.

20. Air dry the pellet for 5–10 min behind the beta shield in the hood. Add 6 μL of water, 6 μL of 100% formamide and vortex to dissolve the pellet. Briefly centrifuge the tubes to bring down sample to the bottom of the tube.
21. Heat samples at 95°C for 10 min before loading on to the gel.

**Pause point:** DNA samples after UVDE digestion and ethanol precipitation can be stored at −20 before heating. It is recommended to heat the samples immediately before loading.

**CRITICAL:** Use lid locker or a heated lid equipment so that the heated radioactive sample does not pop out of the tube during this step.

### Prepping and assembly of glass plates

**Timing: 15–30 min**

This is a preparation step for casting the denaturing urea polyacrylamide gel. A neat and clean assembly of glass plates facilitates smooth casting of the gel. (Sequi-Gen GT Nucleic Acid Electrophoresis Cell Instruction Manual)

22. Lay the outer long and inner short IPC glass plates flat on the table surface supported by two pipette tip boxes. Using a Kimwipe, wipe the surface of Sequi-Gen GT glass plates with water, then with ethanol. Let it dry.
  a. Add 2–3 drops of Rain-X to one side of the outer (long) glass plate and spread it evenly throughout the glass plate using a clean Kimwipe. This will appear as a thin film of coating on the glass plate when it is wet. This coated side will be in contact with the gel.
    ***Note:*** Excess RainX should be cleaned from the plate. Otherwise, this leads to floating of gels while running the gel.
  b. Wipe the spacers (0.17 mm) with ethanol and place one on each side of the integral plate chamber (IPC) glass plate. Now place the long outer glass plate over the spacers. Lift the whole set up and place vertically onto a clean surface and insert the GT lever clamps on each side one after the other. The clamps must be tight enough to avoid any leakages. Place the whole set up into the precision caster base and loosen the precision caster cam pegs so that the precision cam pegs fit properly into the holes of the side clamp. Tighten the precision caster cam pegs and lay the whole set up flat on the bench with the upper part a few inches raised. One could use a 15 mL centrifuge tube for the raised support.

### Preparation and casting of denaturing urea polyacrylamide gel

**Timing: 1 h 45 min**

A denaturing 15% polyacrylamide urea gel is casted in this step.

23. Add the specified ingredients in a 200 mL beaker with stir bar to make the urea gel solution.

**Table:** 

Reagent	Amount
40% Acrylamide	26.25 mL
10 × TBE	7 mL
ddH_2_O	14.75 mL
Urea	29.7 g
10% APS	0.75 mL
TEMED	0.02 mL

24. Place the beaker on a magnetic stirrer and let it stir until urea dissolves completely.
  a. After urea dissolves completely, place the beaker on ice until you are ready to inject the solution into the gel apparatus. This step avoids quick/earlier solidification of gel before injection or during injection into the gel apparatus.

**CRITICAL:** Acrylamide is a neurotoxin and can be absorbed through skin. Unsolidified acrylamide should be used with caution during this step to avoid spills. Allot separate space for casting gel. Wear PPE to avoid acrylamide getting into eyes or skin.

25. Right before casting the gel, add 750 μL of 10% APS and 20 μL of TEMED to the gel solution and mix slowly and gently, avoiding formation of bubbles.
  a. Suck up the solution gently using the Luer taper attached to a 60 mL syringe. It is critical to avoid bubbles at this step. If there are any bubbles formed, make sure to get rid of them (by gentle tapping and injecting the bubble out of the syringe and Luer taper) before injecting the solution into the gel apparatus.
26. Slowly inject the gel solution. Once the glass plates are filled with the gel solution, insert the comb between the glass plates.
  a. While injecting and fixing the comb, be gentle to avoid any air bubbles at the interface between gel and comb. Otherwise, wells might get uneven at the bottom after solidification. Make sure to use combs and spacers of same thickness.
27. Let the gel solidify for at least 1 h.
  a. The gel can be left to solidify for up to 16 h. The edges can be covered with wet paper towel soaked in 1× TBE and then covered with saran wrap and stored flat at 4°C (up to a few days but not more than a week).

### Pre-running, sample loading and electrophoresis

**Timing: 3 h and 15 min**

UVDE digested samples are loaded and electrophoresis at high voltage current is carried out at this step.

28. Set up the gel assembly behind a beta shield. Do not take out the gel plates from behind the shield once the radioactive sample is loaded on to the gel.
  a. The gel assembly is placed inside the GT base buffer tank and the glass plates are supported with GT bottom safety cover that has screws to be tightened to secure the vertical glass plates to GT base.

***Note:*** Refer to Sequi-Gen® GT Nucleic Acid Electrophoresis Cell instruction manual for complete details (Sequi-Gen GT Nucleic Acid Electrophoresis Cell Instruction Manual)

29. Pour 1× TBE buffer into the buffer chamber at the bottom and the inner buffer chamber attached to the lPC shorter glass plate.
  a. Flush the wells with buffer using a gel loading pipette.
  b. Cover the inner buffer chamber with GT top safety cover so that the electrodes on the GT lever clamps are connected to the electrodes on the GT top safety cover.
    **CRITICAL:** Use caution while handling high voltage powerpack. Leakage of buffer is possible during the run. Make sure the GT lever clamps are fit snuggly to avoid buffer leakage. Monitor gel electrophoresis few times during the run to check for buffer leakage.
  c. Connect the system to a high voltage powerpack. Pre-run the gel at 60 watts for 30–40 min or until the thermosensor tape on the glass plates shows the temperature between 40°C–50°C. Turn off the powerpack, take off the lid, and flush the wells gently once again to get rid of urea.
    **CRITICAL:** Every step after this is carried out safely behind a beta shield. Be cautious and gentle while handling radioactive samples and gels. Avoid cluttering of the bench space where radioactivity is handled.

30. Add 12 μL of the heated samples to the wells. Leave one well empty between marker and samples. 10 μL of diluted marker oligos can be added to the wells. Heating of marker oligos is not necessary.
  a. It is recommended to check the radioactivity of samples and marker oligos using Geiger Muller counter. Total radioactivity of markers should be 3 to 5 times less than the total radioactivity of samples. Dilute the marker oligos using water to adjust for ideal radioactivity signal. This helps reduce the signal intensity of markers taking over the signal intensity of samples while imaging. We do not add loading dye to samples and marker oligos but adding loading dye to empty wells helps track the gel running. Without loading dye it is hard to determine how far the gel has run.
  b. Electrophoresis is carried out at 60 watts for 2 h and 10 min. At this time point the dye should have separated into upper and lower band. The lower band should reach approximately 10–15 cm from the bottom of the glass plates.
  c. Turn off the high voltage current and remove the GT top safety cover lid disconnecting the electrodes. Carefully pour the buffer into a wide mouthed radioactive waste beaker.
  d. Using a separator, try to gently separate the glass plates such that the gel sticks to the bottom plate. Gently flip the glass plates if the gel is sticking on to the upper plate.
  e. Cover the gel with saran wrap.

### Exposure to phosphor screen and imaging

**Timing: at least 2 h and 30 min (might take longer exposure h depending upon the signal intensity of the sample)**

The urea polyacrylamide gel is exposed to a phosphor screen and the signal is captured using a typhoon scanner. The image is quantified using Imagequant software.

31. Place phosphor screen on top of the gel covered by saran wrap so that the white portion (phosphor side) is facing the gel and leave the phosphor screen undisturbed for 2 h.

**CRITICAL:** Carefully discard the radioactive gel into appropriate discards. Save the gel until image is scanned.

**Figure 3:**
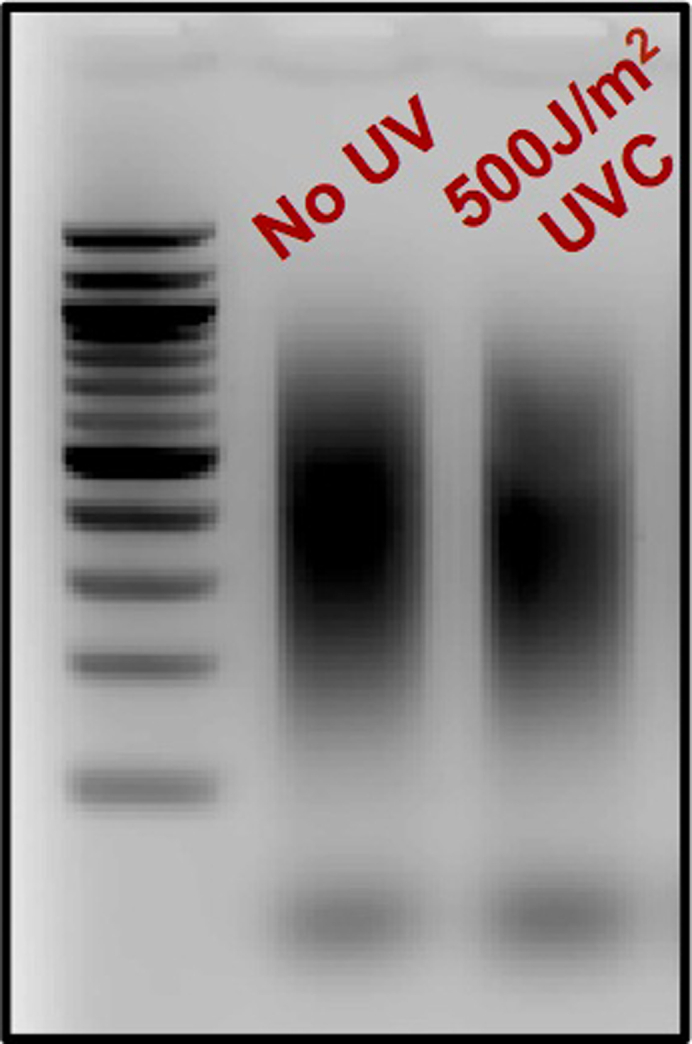
Agarose gel confirmation of proper sample sonication

***Note:*** Refer to Typhoon™ FLA 7000 user manual for complete details on using phosphor stage and scanning procedure (Typhoon FLA 7000 User Manual).

32. Take magnetic phosphor stage near the phosphor screen. Once the phosphor screen sticks to stage adjust phosphor screen to center of the platform within the enclosed raised frame.
  a. If the screen is not properly aligned within the enclosed frame, there are chances of the screen getting stuck or slipped during scanning process in typhoon scanner
33. Place the magnetic platform onto Typhoon scanner such that the pegs on the typhoon scanner fit into holes of the magnetic platform.
  a. Appropriate alignment is essential at this step otherwise the platform will get stuck during the scanning process.
34. Scan image using typhoon scanner phosphorimaging software. Save the image and analyze using ImageQuant software. Lanes were manually created and spots corresponding to ‘TA’ lesions were quantified and exported to excel. The values obtained were used to make the graph shown in Figure 3.

### *In vivo* UV-C damage induction

**Timing: about 8 h**

The purpose of this step is to induce sufficient numbers of 6,4-PPs and atypical UV lesions to be mapped. A UV-C dose between 500 and 600 J/m^2^ will induce approximately one 6,4-PP per 1 kb yeast DNA.

35. Grow yeast cells in yeast extract peptone dextrose (YPD) medium for 16 h and subculture the yeast cells in 40 mL (20 mL per timepoint) YPD. Incubate the cells at 30°C to an OD_600_ of about 0.8 (between 0.4–1.0).
  a. Subculture growth will take around 6 h, depending on the strains being used.
  b. Pellet cells at 3200×*g* for 5 min, remove media, and resuspend in 40 mL ddH_2_O.
    i. Exposing cells suspended in colored media such as YPD to UV-C will result in reflection of UV off the media, and thus a lower UV dose than intended.
36. Aliquot 20 mL of cells for “No UV” samples, centrifuge, remove the medium, and store the cell pellet at −80°C.
37. In a dark room, pour remaining 20 mL of cells into a 5” × 7” plastic developing tray (or other container that will allow for ample spread of cells). Expose cells to 500–600 J/m^2^ UV-C (based on manufacturer’s calculations) with the container lid off.
  a. Immediately transfer irradiated cells to a conical tube, pellet cells as described above, and remove supernatant.
    i. It is recommended that gDNA be isolated immediately. However, cells may be stored at −80°C until gDNA isolation if necessary.
38. For UV-exposed naked DNA control libraries, grow cells as described above, but do not expose to UV-C light.
  a. Following gDNA isolation (described in next section), resuspend pellet in 100 μL ddH_2_O.
  b. Spot DNA onto microscope cover slip in 10 μL aliquots.
    i. Expect to recover about 80% of starting volume.
    ii. If sample is spotted onto a microscope slide it will spread out and will not be able to be recovered.
  c. Place cover slip onto a dish filled with ice. This will help combat sample evaporation.
  d. Expose sample to 400–500 J/m^2^ UV-C. The UV dose of naked DNA samples should be approximately 80% of the cellular dose.
    i. Following UV exposure, pipette samples off of cover slip and combine in 1.5 mL microcentrifuge tube.
    ii. Bring total volume up to 310 μL with dH_2_O. Samples can be taken to sonication as described below.

### Genomic DNA (gDNA) isolation

**Timing: About 20 h**

The purpose of these steps is to extract whole genomic DNA from UV-irradiated yeast for subsequent lesion mapping using UVDE-seq.

39. Let cells thaw at 23°C.
  a. Rinse cells with ddH_2_O to remove residual media.
  b. Add 250 μL yeast lysis buffer to cell pellet. Resuspend cells and transfer to a 1.5 mL microcentrifuge tube.
  c. Add approximately 150 μL acid-washed glass beads and 250 μL PCI (25:24:1, pH ∼6.7). Vortex at highest setting for 4 min.
    i. Following vortex, endogenous photolyase activity is killed and samples are safe to be in light.
  d. Add 200 μL TE buffer pH 8 and vortex for 1 min.
  e. Centrifuge for 10 min at minimum 15,871×*g*.
  f. Transfer the clear aqueous layer to a fresh tube and precipitate the DNA by adding 1 mL 100% cold Ethanol. Incubate for 3–5 min at 23°C and centrifuge for 3 min at minimum 15,871×*g*.
    i. A pellet should be seen at the bottom of the tube.
  g. Remove the supernatant mixture of TE buffer and 100% Ethanol and rinse pellet with 500 μL 75% Ethanol. Centrifuge for 3 min at minimum 15,871×*g*.
  h. Remove 75% Ethanol and briefly spin the tube to pull excess Ethanol to the bottom of the tube. Pipette off remaining Ethanol.
  i. Air dry pellet for approximately 5 min, or until remaining Ethanol has evaporated.
  j. Dissolve the gDNA pellet in 200 μL TE buffer pH 8 by pipetting and/or vortexing.
  k. Add 2 μL RNase A. Tap tube to mix and incubate at 37°C for 30–60 min
    i. 60 min helps to reduce smaller bands on gel.
  l. Add 200 μL PCI (25:24:1, pH ∼6.7) and vortex for 30 s.
  m. Add 200 μL TE buffer pH 8 and invert tube several times to mix.
  n. Centrifuge for 5 min at minimum 15,871×*g*.
  o. Transfer aqueous layer to a new 1.5 mL microcentrifuge tube.
    i. Add 1/10^th^ total volume 3M NaOAc and flick to mix.
    ii. Add 2 volumes of cold 100% Ethanol and invert to mix.
      **Pause point:** Incubate at −20°C for at least 15 min, but preferably 16 h.
  p. Centrifuge for 30 min at 15,871×*g* in 4°C microcentrifuge. Discard supernatant.
  q. Wash pellet with 500 μL cold 75% Ethanol. Centrifuge for 5 min at 15,871×*g* in 4°C microcentrifuge.
    i. Remove supernatant, then briefly spin down tube to pull down and remove excess Ethanol.
    ii. Air dry pellet for approximately 5 min, or until remaining Ethanol has evaporated.
  r. Resuspend pellet in 310 μL ddH_2_O.
  s. If creating a naked DNA library, take the samples to UV-C exposure as described above in UV-C damage induction.

### Sonication and ethanol precipitation

**Timing: About 20 h**

Sonication allows for the fragmentation of genomic DNA into approximately 200–700 bp in length.

40. Transfer 310 μL sample to a TPX tube.
41. Sonicate using BioRuptor for 15 cycles, 30 s on/off on the HIGH setting.
42. Confirm fragment size distribution by running a 2% agarose gel for 20 min at 120V (Figure 3).
  a. Run the following: 5 μL sonicated DNA sample + 1 μL 6× purple loading dye and 3 μL of 20 ng/μL 100 base pair DNA ladder + 2 μL ddH_2_O + 1 μL 6× purple loading dye.
  b. Image agarose gel on Typhoon using Cy2 filter to verify that DNA fragments range from 200–700 bp in length.
43. Following agarose gel confirmation, transfer samples from TPX tubes to 1.5 mL microcentrifuge tubes.
  a. Add 2 μL glycogen, 30 μL 3M NaOAc, and 850 μL 100% cold Ethanol to each tube, flicking to mix between addition of each reagent.
  b. Incubate tubes at −80°C for at least 1 h or −20°C for 16 h.

**Pause point:** You may incubate samples at −20°C for 16 h and resume protocol the following day.

**Figure 4:**
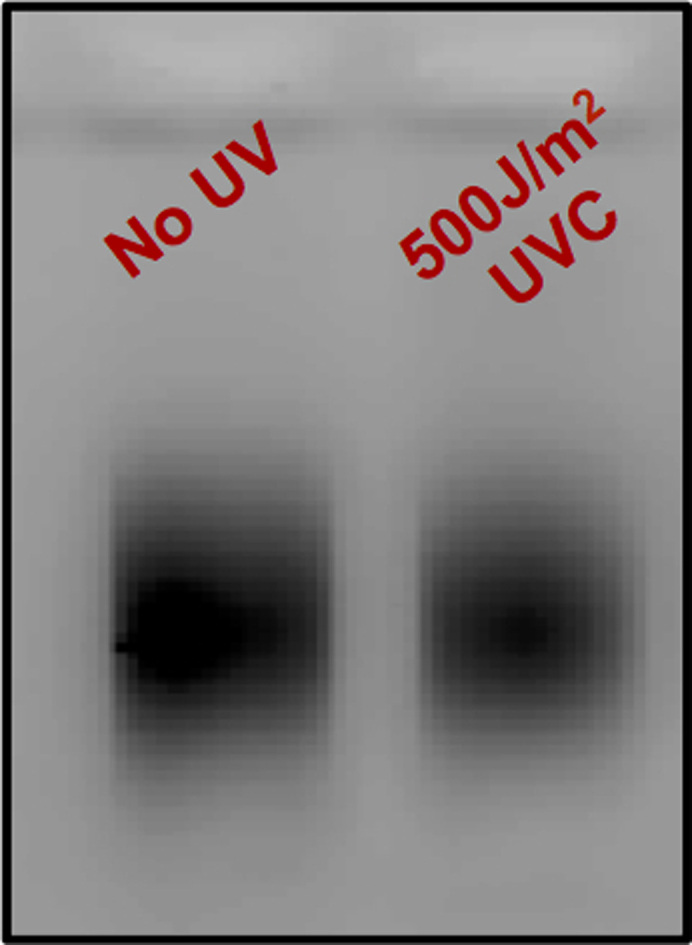
Agarose gel confirmation of sample standardization

44. Centrifuge for over 1 h at 15,871×*g* at 4°C to pellet DNA.
45. Discard supernatant and wash pellet with 500 μL cold 75% Ethanol.
  a. Centrifuge for 30 min at 15,871×*g* at 4°C.
  b. Remove Ethanol, then briefly spin tube to pull down and remove excess Ethanol.
46. Air dry pellet for approximately 5 min, or until remaining Ethanol has evaporated.
47. Dissolve pellet in 110 μL ddH_2_O by pipetting and/or vortexing.
48. If standardization among samples is necessary, measure DNA concentration of each sample using a spectrophotometer. Samples should be standardized to the UV-exposed sample with the lowest DNA concentration. Determine the amount of each sample to carry forward, bringing the final volume of each sample up to 110 μL.
  a. To verify standardization, dilute 5 μL of sample into 20 μL ddH_2_O (1:5 dilution) and run 5 μL of each sample on a 2% agarose gel (Figure 4).

49. Purify DNA by adding 1.2× volume (132 μL) of AMPure XP beads to sample.
  **CRITICAL:** Vortex or resuspend the AMPure XP beads prior to use.
  a. Vortex, then allow to incubate on bench for 5 min.
  b. Place tubes on magnet rack. Allow beads to move to the magnet (approximately 2 min) and remove supernatant.
  c. Wash beads twice with 500 μL of cold 75% Ethanol.
    i. Pipette off Ethanol.
    ii. Remove tubes from magnet and briefly spin tube to pull down excess Ethanol that may remain on the sides of the tubes. Place tubes back on magnet rack. Pipette off remaining Ethanol.

Performing this additional spin helps ensure excess Ethanol is not left behind in the tube and carried forward through subsequent steps.

d. Air dry beads for approximately 5 min, or until beads appear to be slightly dry and have a faintly cracked surface.
  **CRITICAL:** Do not over dry beads
e. Resuspend beads in 86 μL ddH_2_O by vortexing. Incubate at on bench for 5 min.
f. Place tubes on magnet rack and transfer supernatant to a fresh 1.5 mL microcentrifuge tube.
  ***Optional:*** Run 5 μL of 1:5 diluted sample on a 2% agarose gel to confirm standardization and AMPure bead purification. Make sure intensity of samples are within 15% intensity of each other.

### End repair, dA-tailing, and first adapter ligation

**Timing: 4+ h**

This step aims to eliminate overhangs that may be created during sonication and ligate the trP1 adapter to the 3′ end of the DNA fragments.

50. Add 10 μL 10× buffer and 5 μL of enzyme mix from NEBNext end repair module. Incubate at 20°C for 2 h.
51. Purify DNA by adding 1.2× volume of AMPure XP beads (120 μL) to each tube, briefly vortex to mix, and incubate at 23°C for 5 min.
  a. Place tubes on magnet rack. Allow beads to move to the magnet (approximately 2 min) and remove supernatant.
  b. Wash beads twice with 500 μL of 75% cold Ethanol.
    i. Pipette off Ethanol.
    ii. Remove tubes from magnet and briefly spin tube to pull down excess Ethanol. Place tubes back on magnet rack. Pipette off remaining Ethanol.
  c. Allow beads to air dry on bench (approximately 5 min).
  d. Resuspend beads in 42 μL ddH_2_O by vortexing to mix.

**CRITICAL:** Do not remove beads

52. Add 5 μL of 10× buffer and 3 μL of Klenow fragment (3’->5′ exo) from NEBNext dA-tailing module. Incubate tubes at 37°C for 1 h.
  a. To purify DNA, add 17 μL 5M NaCl and 22.5 μL of 40% PEG 8000 (1.25M NaCl), and vortex to mix.
  b. Place tubes on magnet rack. Allow beads to move to magnet and remove supernatant.
  c. Wash beads twice with 500 μL of 75% cold Ethanol.
    i. Pipette off Ethanol.
    ii. Remove tubes from magnet and briefly spin tube to pull down excess Ethanol. Place tubes back on magnet rack. Pipette off remaining Ethanol.
  d. Air dry beads for approximately 5 min, or until beads appear to be slightly dry and appear to have a cracked surface.
  e. Resuspend beads in 20 μL ddH_2_O by vortexing to mix.

**CRITICAL:** Do not remove beads

53. Add 10 μL NEB 5× buffer, 15 μL of 40 μM double-stranded dstrP1 adapter, and 5 μL NEB quick ligase, flicking to mix. Incubate mixture at 20°C for 2 h (preferred) or 16°C for at least 12 h.

**Pause point:** You may choose to ligate at 16°C for at least 12 h and resume the protocol the following day.

### First adapter confirmation

**Timing: 1.5 h**

Before continuing, it is important to check the success of your first adapter ligation. Successful ligation of the first (trP1) adapter will result in ample signal of PCR product (appears as a smear) using the trP1 primer. It is possible for signal of the UV-exposed sample to appear lower than that of the unexposed samples. We believe this is due to polymerase struggling to process through UV damage.

54. To the first adapter ligation reaction, add 17 μL 5M NaCl and 22.5 μL of 40% PEG8000 (1.25M NaCl), vortexing to mix.
  a. Place tubes on magnet rack. Allow beads to move to the magnet and remove supernatant.
  b. Wash beads twice with 500 μL of cold 75% Ethanol.
    i. Pipette off Ethanol.
    ii. Remove tubes from magnet and briefly spin to pull down excess Ethanol. Place tubes back on magnet rack. Pipette off remaining Ethanol.
  c. Air dry beads for approximately 5 min, or until beads appear to be slightly dry and appear to have a cracked surface.
    **CRITICAL:** Do not over dry beads
  d. Resuspend beads in 100 μL ddH_2_O. Vortex to mix and incubate at 23°C for 5 min.
  e. Place tubes on magnet rack and transfer supernatant to a fresh 1.5 mL tube.

55. Take measurement of samples using a NanoDrop spectrophotometer. Samples must total 50 μg or less.

56. Perform PCR to confirm first adapter ligation using volumes listed below (20 μL reaction volume).
  **Table:** 

Reagent	1× Vol. (μL)
Taq Buffer	2
3 mM DNTPs	1
5 μM trP1 Primer	1
H2O	14.85
DNA Sample	1
Taq polymerase	0.15
  a. PCR should follow steps shown below.
    **Table:** 

Steps	Temperature	Time	Cycles
Initial denaturation	94°C	3 min	1
Denaturation	94°C	30 s	5–9 cycles
Annealing	55°C	30 s	
Extension	72°C	30 s	
Final extension	72°C	1 min	1
Hold	4°C	Forever

57. Run 10 μL on a 2% agarose gel at 120 V for 20 min (Figure 5).
  a. Image on Typhoon using Cy2 filter.

**Figure 5:**
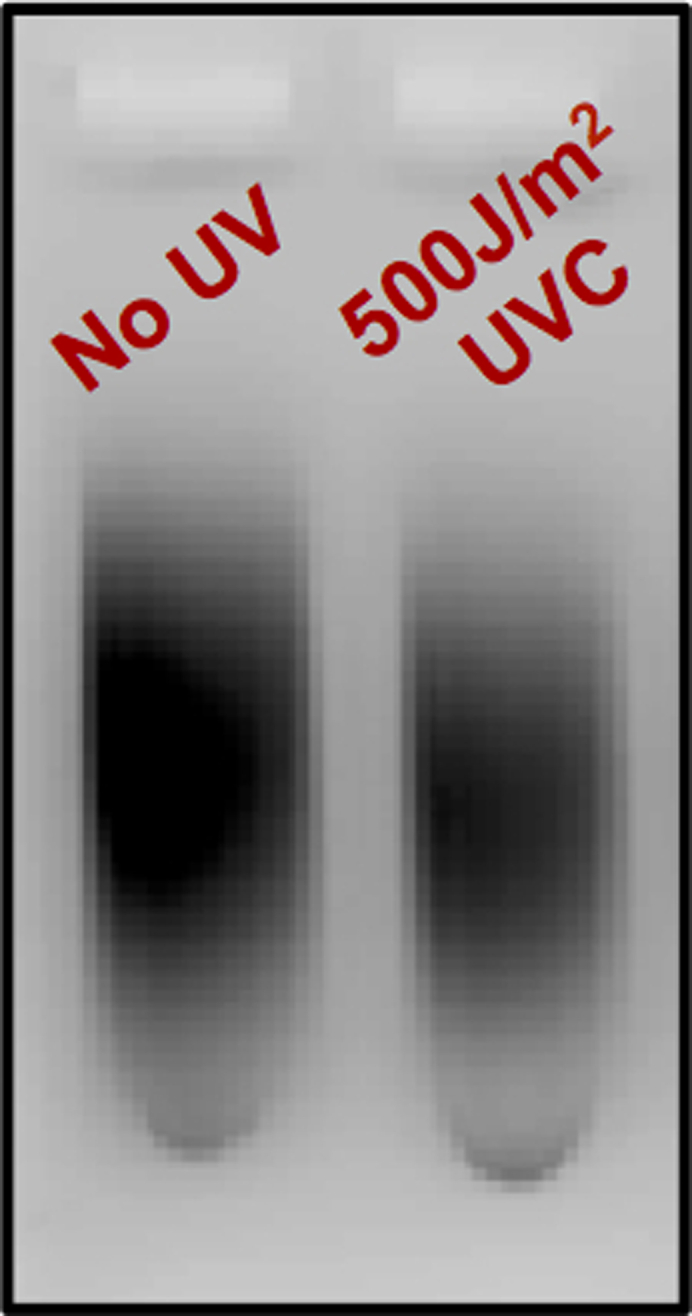
Agarose gel confirmation of first adapter ligation
58. Following PCR verification, bring volume up to 195 μL with ddH_2_O.

### CPD photolyase treatment and UVDE cleavage

**Timing: about 9 h**

These steps aim to repair CPD lesions, leaving behind only (6–4)PP and atypical lesions that are subsequently cleaved by UVDE. Cleavage by UVDE creates a 3′ OH group just upstream of the lesion, which allows for second (A) adapter ligation.

59. Add 25 μL NEB 10× terminal transferase buffer, 2.5 μL 10 mM ddATP or ddGTP, 25 μL 2.5 mM CoCl_2_, and 2.5 μL 20 U/μL NEB terminal transferase, flicking to mix between each reagent. Incubate at 37°C for 2 h.
60. Add 1.2× volume of AMPure XP beads (300 μL) to each tube, briefly vortex to mix, and incubate at 23°C for 5 min.
  a. Place tubes on magnet rack. Allow beads to move to magnet and remove supernatant.
  b. Wash beads twice with 500 μL of cold 75% Ethanol.
    i. Pipette off Ethanol.
    ii. Remove tubes from magnet and briefly spin tube to pull down excess Ethanol. Place tubes back on magnet rack. Pipette off remaining Ethanol.
  c. Air dry beads for approximately 5 min, or until beads appear to be slightly dry and have a faintly cracked surface.
  d. Resuspend beads in 25 μL ddH_2_O. Vortex to mix. Let sit at 23°C for 5 min.
  e. Place tubes on magnet rack. Allow beads to move to magnet and move supernatant to a clean 1.5 mL tube.
61. Add 3 μL CPD photolyase buffer and 2 μL CPD photolyase to each tube. Incubate at 23°C under 365 nm UV light for 2 h.
  a. If using a hand-held UV light, you may place samples under a glass pyrex dish and set the light on top of the Pyrex.
    i. The distance at which the hand-held UV light should be placed above the sample is based on the manufacturer’s recommendation and should be verified using a radiometer. Additionally, conditions should be optimized to ensure repair of all CPD lesions.
62. Following incubation, add 30 μL PCI (25:24:1, pH ∼6.7) and vortex about 30 s.
  a. Add 30 μL TE buffer pH 8 and invert 3 times.
  b. Centrifuge samples at 15,871×*g* for 10 min in 4°C microcentrifuge.
  c. Transfer aqueous layer to new tube.
  d. Add 1.5 μL glycogen, 6 μL 3M NaOAc (or 1/10^th^ final volume), and 150 μL–200 μL cold 100% Ethanol. Incubate at −80°C for at least 1 h.
  e. Following incubation, centrifuge samples at 15,871×*g* for 15 min in 4°C microcentrifuge.
  f. Remove supernatant and wash pellet with 400 μL cold 75% Ethanol. Spin at 15,871×*g* for 5 min in 4°C microcentrifuge.
  g. Remove Ethanol and dry pellet near flame for about 5 min until all Ethanol has evaporated.

63. Resuspend pellet in 21 μL ddH_2_O. Add 6 μL 10× UVDE buffer and 3 μL UVDE. Incubate at 55°C for 45 min.
  **CRITICAL:** move quickly after this step!
  a. Add 1.2× volume (36 μL) of AMPure XP beads to sample. Allow to incubate on bench for 5 min.
  b. Place tubes on magnet rack. Allow beads to move towards magnet and remove supernatant. Wash beads twice with 500 μL of cold 75% Ethanol.
    i. Pipette off Ethanol.
    ii. Remove tubes from magnet a briefly spin tube to pull down excess Ethanol. Place tubes back on magnet rack. Pipette off remaining Ethanol.
  c. Air dry beads for approximately 5 min, or until beads appear to be slightly dry and appear to have a cracked surface.
  d. Resuspend beads in 85 μL ddH_2_O. Vortex to mix.
    **CRITICAL:** Do not remove beads

### Dephosphorylation and second adapter ligation

**Timing: About 14 h**

These steps aim to dephosphorylate the 5′ ends of the DNA fragments to avoid self-ligation and circularization of samples, and ligate the second (A) adapter to the 3′ end.

64. Add 10 μL of 10× SAP (Shrimp Alkaline Phosphatase) buffer to each tube, flicking to mix. Add 5 μL of 1 U/μL SAP to each tube, flicking to mix. Incubate at 37°C for 1 h, followed by 65°C for 15 min to heat kill the reaction.
  a. Add 34 μL NaCl and 45 μL 40% PEG8000 (1.25M NaCl) to each tube, briefly vortexing to mix.
  b. Place tubes on magnet rack. Allow beads to move to magnet and remove supernatant.
  c. Wash beads twice with 500 μL of 75% cold Ethanol.
    i. Pipette off Ethanol.
    ii. Remove tubes from magnet a briefly spin to pull down excess Ethanol. Place tubes back on magnet rack. Pipette off remaining Ethanol.
  d. Air dry beads for approximately 5 min, or until beads appear to be slightly dry and appear to have a cracked surface.
    **CRITICAL:** Do not over dry beads
  e. Resuspend beads in 29 μL ddH_2_O by vortexing to mix.
    i. Incubate on bench for 5 min.
    ii. Pellet beads using the magnet rack and transfer supernatant to a new tube.

65. Incubate tubes at 95°C for 5 min in thermal cycler to denature DNA.
  a. Immediately transfer to ice to snap cool.
66. Add 10 μL NEB 5× buffer, 6 μL 40 μM dsA adapter (adapters A1-A6, one per sample), and 5 μL NEB T4 quick ligase to each tube and incubate in thermal cycler at 20°C for 2 h OR at 16°C for at least 12 h.

**Pause point:** You may choose to ligate at 16°C for at least 12 h and resume protocol the following day.

### Second adapter confirmation

**Timing: 1.5 h**

Before continuing with library preparation, it is important to verify the success of your second adapter ligation. Successful ligation of the first (trP1) adapter will result in signal of PCR product (appears as a smear) using the Cy3-labelled A primer.

67. Purify DNA by adding 1.8× volume (90 μL) of AMPure XP beads to sample. Allow to incubate at 23°C for 5 min.
  a. Place tubes on magnet rack. Allow beads to move to magnet and remove supernatant.
  b. Wash beads twice with 500 μL of 75% cold Ethanol.
    i. Pipette off Ethanol.
    ii. Remove tubes from magnet rack and briefly spin tube to pull down excess Ethanol. Place tubes back on magnet rack. Pipette off remaining Ethanol.
  c. Air dry beads for approximately 5 min, or until beads appear to be slightly dry and appear to have a cracked surface.
  d. Resuspend beads in 40 μL ddH_2_O by vortexing to mix.
  e. Incubate on bench for 5 min. Pellet beads using the magnet rack.
68. Transfer supernatant to a new tube. Run PCR to check second adapter ligation, using the reagents listed below and the reaction steps previously described in the ‘end repair, dA-tailing, and first adapter ligation’ section.

**Table:** 

Reagent	1× vol. (μL)
Taq buffer	2
3 mM dNTPs	1
5 μM trP1 primer	1
5 μM Cy3 primer A	1
H_2_O	15
DNA	1
Taq polymerase	0.15

69. Following PCR, add 5.3 μL of 50% glycerol to each PCR tube (final glycerol concentration will be 10%).
  a. Load 10 μL of each sample and run on 2% agarose gel without any gel dye at 120 V for 20 min.
  b. Image on Typhoon using Cy3 filter.
  c. You should expect to see more signal in the UV-exposed sample compared to the unexposed sample.

### Single-stranded library preparation

**Timing: about 13 h**

Brief description: These steps allow for the removal of the second strand of DNA that does not contain the lesion of interest, leaving a single stranded DNA template to be PCR amplified and sequenced.

70. Add 20 μL Dynabeads M-280 Streptavidin per sample to a fresh 1.5 mL microcentrifuge tube and place it on the magnet rack.
  a. Discard the supernatant.
71. Add 1 mL 1× bind-and-wash buffer to tubes and briefly vortex to mix.
  a. Centrifuge tubes briefly to pull down contents.
  b. Place tubes on magnet rack and discard supernatant.
72. Add 40 μL 2× bind-and-wash buffer and briefly vortex tubes.
  a. Centrifuge tubes briefly to pull down contents.
73. Add samples to tubes with streptavidin beads (entire 40 μL volume).
  a. Vortex tubes briefly and leave on rotator at 23°C for 15 min.
  b. Place tubes on magnet rack and discard supernatant.
74. Add 1× 50 μL saline sodium citrate to each tube and briefly vortex.
  a. Incubate on rotator for 10 min.
  b. Place tubes on magnet rack and discard supernatant.
75. Add 40 μL of 0.15M NaOH to each tube and briefly vortex, then place on rotator for 10 min at 23°C.
  a. Place tubes on magnet rack and transfer supernatant to a new tube.
76. Repeat the NaOH capture and pool with remainder of sample. Add 120 μL ddH2O to tube of pooled eluate from NaOH capture. Add 2 μL glycogen, 20 μL 3M NaOAc, and 550 μL cold 100% Ethanol, flicking to mix between addition of each reagent.
  a. Incubate samples at −20°C for at least 12 h.

### Second strand synthesis

**Timing: 4 h**

Amplification of the single stranded DNA template, using the double stranded A adapter as the primer.

77. Remove samples from −20°C and centrifuge samples for at least 1 h at 15,871×*g* in microcentrifuge at 4°C to pellet DNA.
  a. Discard supernatant and wash pellet with 500 μL cold 75% Ethanol.
  b. Centrifuge for 30 min at 15,871×*g* at 4°C.
  c. Remove supernatant, briefly spin down tube to pull down and remove excess Ethanol.
  d. Air dry pellet for approximately 5 min, or until remaining Ethanol has evaporated.
78. Dissolve pellet in 19.5 μL master mix by pipetting/vortexing. Components of the master mix are listed below.

**Table:** 

Reagent	1× Vol. (μL)
Taq Buffer	2
3 mM DNTPs	1
5 μM Primer A	1
H_2_O	16.85
DNA Sample	-

79. For the Taq polymerase, make a master mix consisting of 8 μL dH_2_O, 1 μL Taq buffer, and 1 μL Taq polymerase. This is enough for 6 samples.
  a. Add 1.5 μL Taq master mix to each PCR tube.
80. Perform PCR as described below.

**Table:** 

Steps	Temperature	Time
Denaturation	98°C	2 min
Annealing	55°C	30 s
Extension	72°C	1 min
Hold	4°C	Forever

81. Purify DNA by adding 1.8× volume (37.8 μL) of AMPure XP beads to sample.
  a. Allow to incubate at 23°C for 5 min.
  b. Place tubes on magnet rack. Allow beads to move to the magnet and remove supernatant.
  c. Wash beads twice with 500 μL of 75% cold Ethanol.
    i. Pipette off Ethanol.
    ii. Remove tubes from magnet and briefly spin tube to pull down excess Ethanol. Place tubes back on magnet rack. Pipette off remaining Ethanol.
  d. Air dry beads for approximately 5 min, or until beads appear to be slightly dry and appear to have a cracked surface.
  e. Resuspend beads in 30 μL ddH_2_O by vortexing to mix and incubate at 23°C for 5 min.
  f. Place tubes on magnet rack and transfer supernatant to a fresh tube.

82. Perform PCR to confirm second-strand synthesis using the volumes specified below.

**Table:** 

Reagent	1× Vol. (μL) trP1 adapter only	1× Vol. (μL) trP1 + A adapter
Taq Buffer	2	2
3 mM DNTPs	1	1
trP1 Primer	1	1
Primer A	-	1
H_2_O	15.85	14.85
DNA Sample	1	1
Taq polymerase	0.15	0.15
  a. One set should contain only trP1 primers (negative control), another should contain both trP1 primers and PrimerA.
  b. The PCR should be run for 8 cycles using the parameters outlined in the ‘end repair, dA-tailing, and first adapter ligation’ section.

83. Following PCR, run 10 μL of each sample on a 2% agarose gel at 120 V for 20 min.
  a. Ample signal should be observed in the set containing both primers, in contrast to the very low signal that should be observed in the set containing one primer.

### Final library amplification and sequencing

**Timing: 1.5 h**

This final step aims to amplify the UVDE-seq library to achieve ample reads for Ion Torrent sequencing. The number of PCR cycles is limited to avoid amplification bias of certain sequences.

84. Perform PCR to amplify library using volumes specified below.

**Table:** 

Reagent	1× Vol. (μL)
Taq Buffer	5
3 mM DNTPs	2.5
trp1 Primer	2.5
Primer A	2.5
H_2_O	29.125
DNA Sample	13
Taq polymerase	0.375

  a. Reaction should run for 8 cycles following steps detailed in the ‘end repair, dA-tailing, and first adapter ligation’ section.

85. Following PCR, add 1.2× volume of AMPure XP beads (66 μL) to each tube.
  a. Allow to incubate at 23°C for 5 min.
  b. Place tubes on magnet rack. Allow beads to move to the magnet and remove supernatant.
  c. Wash beads twice with 500 μL of 75% cold Ethanol.
    i. Pipette off Ethanol.
    ii. Remove tubes from the magnet and briefly spin to pull down excess Ethanol. Place tubes back on magnet. Pipette off remaining Ethanol.
  d. Air dry beads for approximately 5 min, or until beads appear to be slightly dry and appear to have a cracked surface.
  e. Resuspend beads in 30 μL ddH_2_O by vortexing to mix and incubate at 23°C for 5 min.
  f. Pellet beads on magnet rack and transfer supernatant to a fresh tube.
86. Run 5 μL of sample on a 2% agarose gel at 120 V for 30 min. Image gel on Typhoon using Cy2 filter (Figure 6).

**Figure 6:**
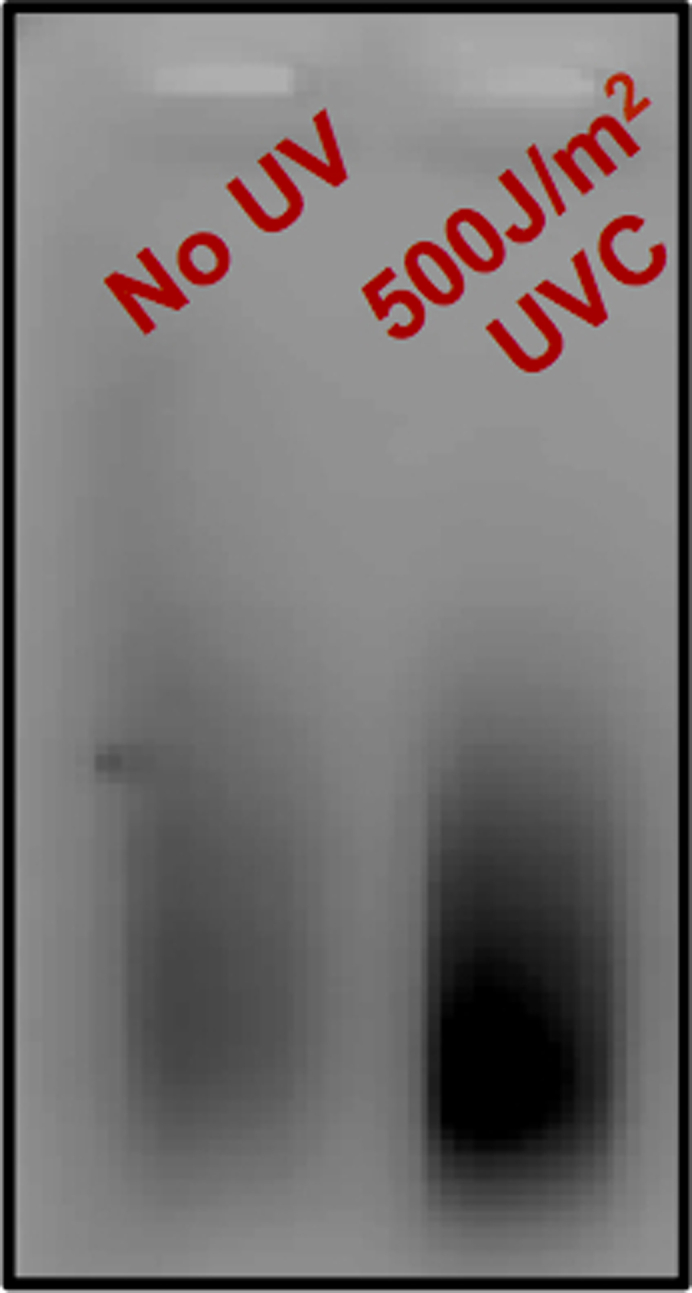
Agarose gel confirmation of final library preparation
87. If final library looks good (signal enrichment in UV-exposed samples), combine 5 μL of each sample into a new 1.5 mL tube and submit for Ion Torrent sequencing.

### Bioinformatic analysis

**Timing: 2 days**

Bioinformatic analysis aims to align sample sequences to the yeast genome (SacCer3) to calculate the abundance of 6,4-PPs and atypical TA lesions throughout. Perl scripts are modified from Ding et al., (2015).

88. Bioinformatic analysis should complete the following tasks:
  a. Separate sequencing reads based on the unique 6 nt barcode sequence associated with each second (A) adapter and remove barcode sequences from fastq file of sequencing reads. Barcode sequences are specified below.

**Table:** 

Adapter	Barcode
A1	AAGAGGAT
A2	TTCGTGAT
A3	CCTGAGAT
A4	ATCGCGAT
A5	TACTGGAT
A6	GAACTGAT
  b. Align reads to the SacCer3 genome using Bowtie2.
    bowtie2 -x saccer3 -U [fastq filename] -S [sam filename]
  c. Convert .sam file to .bam file using Samtools.
    samtools view -b -S [sam filename] >[bam filename]
  d. Convert .bam file to .bed file using bedtools2.
    bedtools bamtobed -i [bam filename] >[bed filename]
  e. Locate the site where damage occurred on the opposite strand of DNA. Example perl script to locate the dinucleotide damage site is shown below.
    while ( my $line = <STDIN> ) {
     my @field = split /\t/, $line;
     if ( $field[5] =∼ s/\+/-/ ) {
     $field[2] = $field[1];
     $field[1] -= 2; }
     else {
     $field[5] =∼ s/-/\+/;
     $field[1] = $field[2];
     $field[2] += 2; }
     if ( $field[1] >= 0 ) {
     print join "\t", @field; } }
  f. Extract dinucleotide sequences for each of the damage sites using bedtools2.
    fastaFromBed -s -name -fi saccer3_genome.fa -bed [damagebed file] -fo [damage fasta file]
  g. Count dinucleotide sequence associated with each lesion for each sequencing library. An example Perl script is given below.
    my %dinuc_count;
    while ( my $line = <STDIN> ) {
     chomp $line;
     if ($line =∼ /ˆ>/ ) {
     next; }
     $dinuc_count{$line}++; }
    foreach my $key (sort keys %dinuc_count) {
     print "$key\t$dinuc_count{$key}\n"; }
  h. Keep only TA lesions by filtering out non-TA lesion sites. An example Perl script to accomplish this is shown below.
    print STDERR "Enter name of bed file:\n";
    my $bedfile = <STDIN>;
    chomp $bedfile;
    open (BED, "$bedfile");
    print STDERR "Enter name of matching dinucleotide sequence file:\n";
    my $dinucfile = <STDIN>;
    chomp $dinucfile;
    open (DINUC, "$dinucfile");
    my $head = "";
    my $dinucseq = "";
    my $match_flag = 1;
    while( my $line = <BED> ) {
     my @field = split /\t/, $line;
     if ( $match_flag ) {
     my $temp = <DINUC>;
     chomp $temp;
     $dinucseq = <DINUC>;
     chomp $dinucseq;
     if ( $temp =∼ /\(/ ) {
     my @fields = split /\(/, $temp;
     $head = $fields[0];
     $head =∼ s/ˆ>//; } }
     if ( $head eq $field[3] ){
     $match_flag = 1;
     if ( $dinucseq =∼ /TA/ ) {
     print $line; } } }
  i. Plot your output (Figure 7) using GraphPad Prism or another software program of your choosing.

**Figure 7:**
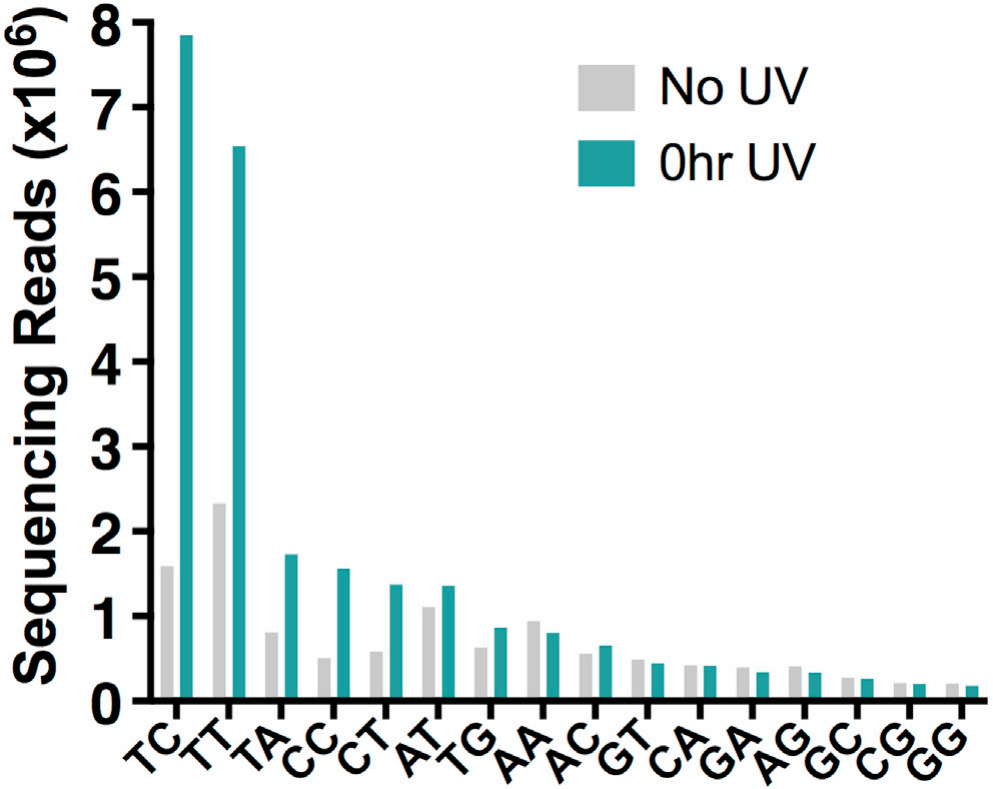
Bioinformatic analysis of lesion distribution (adapted from Laughery et al., 2020)

## Expected outcomes

The success of the *in vitro* validation depends on several factors. First, careful design of the ‘TA’ oligo to induce ‘TA’ photoproduct formation by UV-C light is important. The oligo that we designed here works well for robust induction of ‘TA’ photoproducts (Figures 3A and 3C). Second, selection of the repair enzyme that is able to cleave ‘TA’ photoproducts is critical. Under the conditions we used in this protocol, and as previously suggested in Bryan et al. (2014), the UVDE enzyme ensures optimal cleavage of the ‘TA’ lesion. Failure to observe cleaved products in the denaturing PAGE gel might indicate suboptimal digestion of UVDE that could be attributed to poor quality of the enzyme or one of the buffer components not working well. Finally, a good quality gel image (Figure 3B) largely depends on the signal intensity of ^32^P labelled samples/marker oligos, neutralization of the pH of the UVDE buffer in the samples before loading, and on the electrophoresis running conditions.

The first indication that library preparation was successful will be the agarose gel following the final library amplification (step 86). UV-exposed samples should have visibly more product signal on the gel than unexposed control samples (Figure 6). This will result from more UV lesions being cleaved by UVDE in the UV-exposed samples, which creates the 3′ OH substrate for second adapter ligation. Following library sequencing via the Ion Torrent platform, one can expect anywhere between 17–30 million reads per sample. Bioinformatic analysis must begin by trimming the barcode sequences associated with each adapter. Lack of barcode trimming will result in improper or incomplete sequence alignment. Following analysis outlined in step 55, one can expect to see enrichment of canonical 6–4PPs (TC, TT, CC, CT), as well as noncanonical TA lesions in comparison to unexposed samples (0 h UV compared to No UV sample, Figure 7).

## Limitations

Handling radioactive material (^32^P) needs trained and experienced personnel. Fluorescent labels are not optimal as they might get photobleached differentially for various UV-C doses used in this experiment. The success of each step followed in this protocol cannot be determined until gel imaging on day 3. The quantity of DNA that can be taken through the UVDE treatment may be a limiting factor as well. The UV-C exposure and UVDE cleavage steps were optimized using 50 μg (total) DNA. It is imperative that this quantity is not exceeded, or else UV damage induction and lesion cleavage may be less efficient and ultimately result in decreased enrichment of mappable UV-induced damage.

## Troubleshooting

### Problem 1

Gel breakage during separation of gel from glass plate and during drying is very common when running urea polyacrylamide gels (step 31).

The traditional way is to cut out a filter paper and to place the filter paper on top of the gel. The gel attached to the filter paper is carefully separated from the glass plate. The gel is then covered with a saran wrap.

Place the filter paper side to the gel dryer and turn the heat and vacuum on. Dry the gel for one hour or more until all vapor disappears from the outlet tube attached to the dryer.

Place the dried gel onto a cassette and cover with phosphor screen and expose up to 16 h.

### Potential solution

We have adjusted this protocol to expose the phosphor screen to a wet gel (covered with saran wrap) placed on gel device/ glass plate to avoid the risk of gel breakage while separating gel from the glass plates.

### Problem 2

Radioactive signal of samples/marker oligos is too high or too low (steps 1–5).

### Potential solution

Dilute samples and marker oligos to be less than 100× signal on Geiger Muller counter. When newly labelled marker oligos are not diluted, the signal intensity of the marker oligos will mask the samples while imaging the phosphor screen. Marker oligos should be at least 3–5 times less intense than the samples on a Geiger Muller counter.

If the signal is too low, this may be due to insufficient signal from ^32^P. Check the half-life of ^32^P. Additionally, this may be due to the T4 polynucleotide kinase enzyme and its buffer not working well.

### Problem 3

Failure to see digested products on gel.

### Potential solution

The UVDE enzyme is not functioning well or the buffer and incubation conditions are not suitable for the enzyme action (steps 14–17).

This can also be due to the UV lamp not working sufficiently (step 7).

UV lesion formation and UVDE digestion can be optimized using a control oligo containing CPD-forming dipyrimidine sequences, such as the oligos described in Mao et al. (2018) and Sivapragasam et al. (2021).

### Problem 4

Loss of DNA during AMPure XP bead purification

### Potential solution

AMPureXP bead purification must be carried out very carefully (steps 49, 51, 52, 54, 60, 63, 64a, 67, 81, 85). Do NOT over dry beads. This will result in loss of your library. Following removal of 500 μL 70% Ethanol, briefly spin down tubes to draw down excess Ethanol from the sides of the tube, place back on magnet rack, and using a 10 μL micropipette, pipette off all remaining Ethanol that you can. Next, air dry beads on bench for 5–7 min. Carefully watch beads, and once they appear dry (with small cracks starting to form) add solute. Do NOT exceed 7 min, and do NOT dry beads near a flame. This will over dry the beads and result in a loss of DNA.

### Problem 5

Incomplete sonication.

### Potential solution

Proper sonication is essential to successful Ion Torrent sequencing. If DNA is not fragmented to the appropriate sizes (step 41), one can optimize the number of cycles used for sonication. If DNA fragments are too large (greater than 700 bp) the number of cycles should be increased, and if the fragments are too small (less than 200 bp) the number of cycles should be decreased.

## Resource availability

### Lead contact

Further information and requests for resources and reagents should be directed to and will be fulfilled by the lead contact, John J. Wyrick (jwyrick@wsu.edu).

### Materials availability

This study uses CPD photolyase, which can be purchased from commercial sources, and UVDE, which can be expressed and purified from E. coli, as previously described in Laughery et al. (2020).

## Data Availability

The UVDE-seq data have been submitted to the NCBI Gene Expression Omnibus (GEO; https://www.ncbi.nlm.nih.gov/geo/) under accession number GEO: GSE144679. Code used in this study can be found in the above protocol.
